# Construction and confirmatory factor analysis of the core cognitive ability index system of ship C^2^ system operators

**DOI:** 10.1371/journal.pone.0237339

**Published:** 2020-08-24

**Authors:** Ning Li, Jincai Huang, Yanghe Feng

**Affiliations:** 1 College of Systems Engineering, National University of Defense Technology, Changsha, Hunan, China; 2 Marine Human Factors Engineering Laboratory, China Institute of Marine Technology and Economy, Beijing, China; Univerza v Mariboru, SLOVENIA

## Abstract

**Background:**

Cognitive ability refers to the ability to receive, process, store, and extract information. It is the most important psychological condition for people to successfully complete activities. Previous studies have shown that the design of the human-computer interface of the command and control system cannot exceed the cognitive ability of the operator of the command and control system, and it must match the cognitive ability of the operator in order to reduce the mental load intensity, and improve the accuracy, timeliness and work efficiency. However, previous researchers in the field of cognitive science have not put forward a core index system that can represent the cognitive ability of ship command and control system operators and the importance of each index, and there are few achievements that can be used for reference.

**Objective:**

To explore the core index system of cognitive ability that affecting the cognitive process of command and control system operators, and to verify the index system.

**Methods:**

Based on the classic O*NET questionnaire, two indexes of O*NET were revised, three indexes of response ability were added, and then a questionnaire on the importance evaluation of cognitive abilities index was formed. The questionnaire includes 24 indexes in six aspects: verbal abilities, idea generation and reasoning abilities, quantitative abilities, visual perception abilities, mnemonic and attentive abilities, and response abilities. The cognitive ability importance evaluation data of 202 people from different positions in the ship command and control system were collected. These data reflect the overall level of cognitive ability of operators in the whole ship command and control field.

**Results:**

The data analysis results show that: firstly, the most important cognitive abilities affecting command and control system operators were visual perception abilities, mnemonic and attentive abilities, and response abilities. Secondly, the results of confirmatory factor analysis show that CMIN/DF, GFI, CFI, TLI, RMSEA, RMR and other indicators used in the model test all meet the requirements. The model has a good fitting degree, and the overall index extraction method is feasible. Thirdly, the independence T test results show that for beginners and experienced experts, there is a significant difference in the important evaluation of mnemonic and attentive abilities, while there is no significant difference in the important evaluation of response and visual perception abilities. Fourthly, the results of Bi-group confirmatory factor analysis experiment show that the structural model has good stability and factor invariance.

**Conclusions:**

Through the research of this paper, the index system which can express the core cognitive ability of the commander of command and control system is successfully constructed, and the index system has been fully verified by mathematics. The 3 abilities and 10 indexes in the index system are closely related to the work tasks of operators, which also reflects the correctness of our construction results to a certain extent. According to the results of data analysis, there are differences between assistant commanders and professional commanders in the evaluation of the importance of some indexes, which reflects the importance of working age and experience to the promotion of position skills. The results of this research are of great significance for the subsequent acquisition of cognitive ability data and assessment of post cognitive ability of command and control system operators.

## 1 Introduction

Cognitive ability refers to the ability to receive, process, store, and extract information, which is the most important psychological condition for people to successfully complete activities [1–3]. The abilities of perception, memory, attention, thinking, and imagination are all considered to be cognitive abilities, which are the basis of basic human intelligence activities, as well as necessary conditions for learning, calculation, reasoning and language understanding [4]. Cognitive ability includes verbal abilities, spatial abilities, psychomotor abilities and processing speed abilities [5]. A large number of studies have shown that excellent cognitive ability is a necessary psychological quality for pilots, including good perceptual ability, word memory ability and memory span [6–11]. U.S. army laboratories have proposed that the cognitive abilities and needs of warfighters (commanders) should be fully considered in the design process of military systems, so as to enable users to better use military systems, and this work has become more and more important [12]. The training potential of emerging technologies not only stems from the advanced abilities of the technology, but also from the ability to systematically change teaching methods according to the cognitive needs of learning tasks [13]. FBCB2 system is selected as a tool to identify and record cognitive training technology, which is suitable for typical military digital information systems. FBCB2 is a digital battle command information system used to load leaders and soldiers in the brigade, including all operating systems used in the battlefield [14]. William *et al*. proposed that although modern computers and the internet provide technologically advanced capabilities, in order to stimulate the potential of these systems, cognitive psychology principles and related training techniques need to be integrated into the design of computer training systems [15]. They provided training tables, techniques, outlines and examples for the training system to help troops master training skills and improve the efficiency of skill retention [15]. Deatz suggested that cognitive psychology should be included in the design of future officers and soldiers, and called for the combination of advanced organizers, part-task training, conscious practice, and context-based training to support the future training needs [16].

The above research findings are more about proposing the relevant principles, methods, and training techniques of cognitive psychology should be applied to the design of information systems and military training and provided some training tables, outlines, and examples. However, the above-mentioned cognitive psychology research of information systems and training systems does not propose the extraction method of the user’s core cognitive ability, the composition of cognitive ability index system and the importance of each index. These abilities and corresponding indexes are very important reference bases for military users’ cognitive ability assessment, selection training, and system design. Furthermore, there is a lack of research on the cognitive ability and index system of the operators of the ship command and control(C^2^) system, and there are almost no relevant research results in the existing literature research. Therefore, this paper attempts to study the ship command and control system operators (hereinafter referred to as commanders) core cognitive ability index system construction.

Compared with other studies, the main contributions of this paper are divided into the following four aspects. Firstly, compared with the research of cognitive psychology training principles, methods, techniques and examples in system design and military training, this paper focuses on how to extract the core cognitive ability and corresponding index system of ship command and control commanders. Secondly, this paper provides a complete set of scientific and feasible methods, which can extract the commander’s cognitive ability index system, and solve the problem of lack of relevant methods at present. Three core cognitive abilities of ship commander and corresponding 10 indexes are obtained, which lays the foundation for the follow-up research. Thirdly, the research of this paper is based on the international typical O* NET questionnaire, but it is revised to meet the applicability of the command and control field. In the process of experiment implementation, 202 representatives are selected from multiple positions to carry out the data collection of the cognitive ability index system. Fourthly, descriptive statistics and reliability analysis are used to verify the reliability of the data, exploratory factor analysis (EFA) is used to verify the validity of the data, and confirmatory factor analysis (CFA) is used to verify the accuracy of the data. The results of the above data analysis represent the overall evaluation level of the ship command and control system commander to each cognitive characteristic index.

## 2 Conceptual framework of research

**Fig 1:**
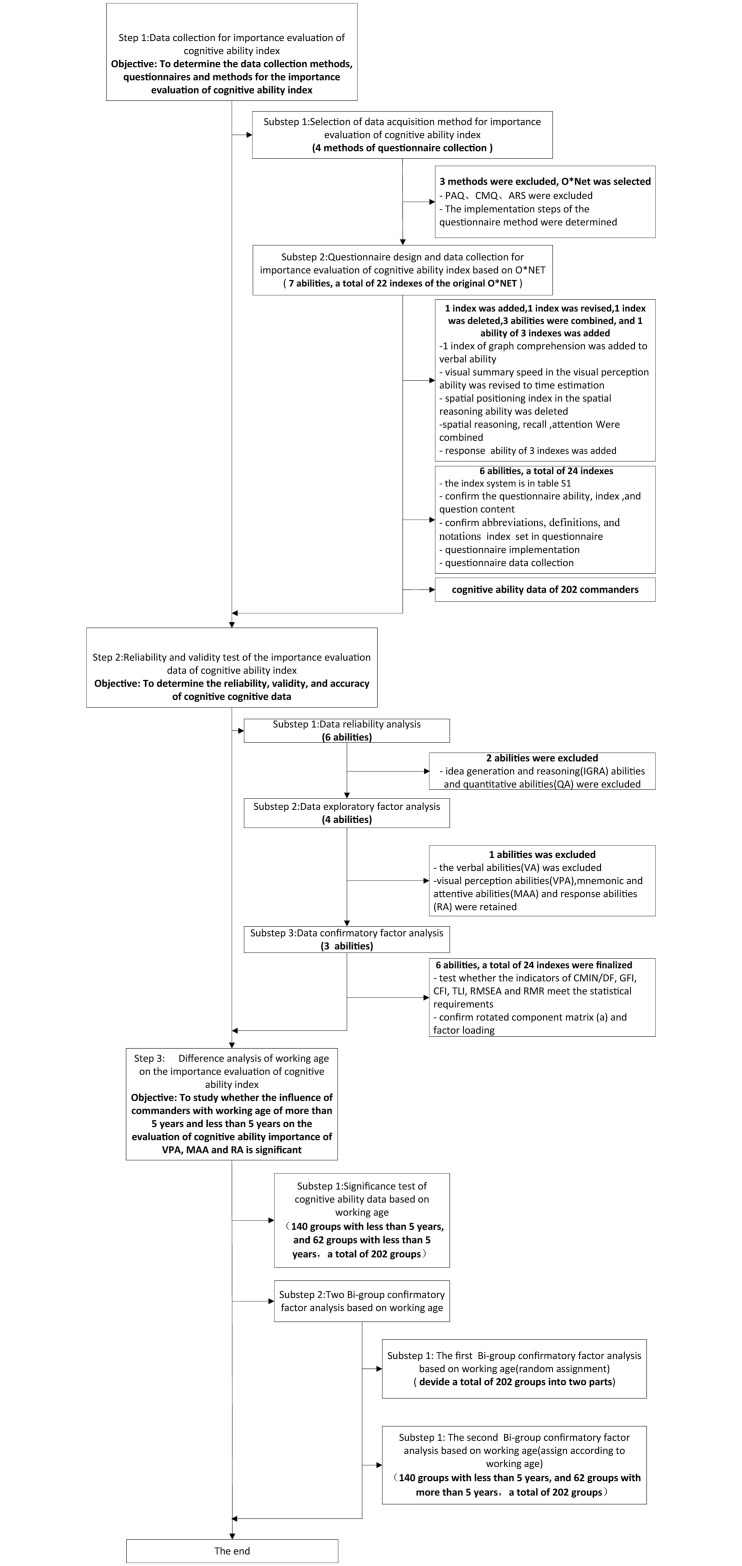
Conceptual model flow chart.

The implementation approach studied in this paper is mainly divided into three steps (see Fig 1 for the detailed process). The first step is to collect the importance evaluation data of cognitive ability index (hereinafter referred to as cognitive ability data). This paper does not involve the data collection of the commander’s cognitive ability itself, but studies the importance of each indicator in multiple cognitive ability indexes. The purpose of this paper is to extract the core cognitive ability index, so it will not be confused with the data collection of the commander’s cognitive ability level.). Firstly, the O* NET questionnaire survey method was selected as the data collection method. Secondly, the ability of data collection questionnaire and corresponding indexes were designed. Finally, the data of the cognitive characteristic index system of 202 people were collected. The 202 subjects were randomly selected from commanders of 4 posts, including situation analysis, task planning, task command and comprehensive support. Their working years range from 1 to 8. During the experiment, we divided them into two categories: one is less than 5 years of working experience (defined as assistant commander), the other is more than 5 years of working experience (defined as professional commander). The second step was to carry out reliability analysis, exploratory factor analysis and confirmatory analysis of cognitive ability data. Firstly, reliability analysis was carried out to determine the reliability of the data and delete the ability that does not meet the requirements. Secondly, exploratory factor analysis was carried out to determine the validity of the data and delete the ability that did not meet the requirements. Finally, confirmatory factor analysis was carried out to verify the accuracy of exploratory factor analysis. The third step was to analyze whether the evaluation importance of the ability and corresponding indexes is different between the subjects with a long working age and those with a small working age.

## 3 Methods and materials

### 3.1 Related principles and methods

The purpose of the command and control system commander’s cognitive ability index system extraction can be directly or indirectly applied to design or evaluation work. Therefore, the principle of cognitive ability index extraction in this paper includes the following three aspects. Firstly, the selected cognitive ability is the key abilities required for commanders to complete the task. Secondly, the selected indexes should be relatively simple and convenient to test, easy to understand, and independent testing without relying on actual equipment. Thirdly, the time of a single test should not be too long, not more than 30 minutes.

The analysis and extraction of cognitive ability index is rooted in the in-depth analysis of task flow and the accompanying cognitive process as well as the collection of cognitive ability data. Cognitive Task Analysis (CTA) is an in-depth understanding of the cognitive processes, cognitive skills, and decision bases in key task activities. It can provide systematic means for targeted selection, training, and system design [17, 18]. In the process of CTA, a variety of methods are required to collect data, including observation method, individual interview, group interview, questionnaire survey, etc. Table 1 lists the definitions, basic operating procedures and advantages and disadvantages of the above four methods.

**Table 1:** Main methods of cognitive task analysis.

Method	Definition	Procedure	Advantage	Disadvantage
individual interview	Collect information by interviewing different people who know the job (e.g. job holder, supervisor)	Interview the job content	More details of information, which can get more information through constant questioning	There may be deviations in the recall of information, and it is difficult to describe the work they do carefully due to the regularization of work
Observation method	Observe how workers complete their tasks, directly or via video	Observe aspects of work through standardized procedures, sometimes in conjunction with thinking aloud to understand the thinking process	Less information deviation, which can find new problems to the greatest extent	Information collection takes a long time, and a standardized expert evaluation system needs to be established
team interview	Set up expert groups to discuss different aspects of the work	Generate work content through brainstorming	Good information consistency, which can minimize the dispersion of information	Information collection is difficult and limited by poor team processes, such as lack of participation of members and herd behavior
questionnaire	Collect structured surveys of job position requirements (pen and paper or computer)	Obtain job content through job analysis questionnaire (PAQ), O*NET method	Large amount of information data, which can collect a large amount of quantitative information	There are many questions that need to be answered in information collection, which may lead to lack of reliability of information

In this project, the research object is the information system commander. Since the commander’s completion of tasks is closely related to the commander’s ability and experience to a large extent, the research scope belongs to the people-oriented work analysis. Personnel oriented job analysis methods include Position Analysis Questionnaire (PAQ) [19], Critical Incident Technique (CIT) [20], Job Element Method (JEM) [21], Behavior Consistency Method (BCM), Threshold Traits Analysis (TTA) [22], and Ability Requirements Scale (ARS), Occupational Information Network (O*NET) [23], among which CIT, JEM and BCM research methods are more complex and difficult to implement, so it is not suitable to conduct large-scale data collection for command and control system commanders. Taking into account the actual working environment, various posts and work contents of the commander’s long-term voyage at sea, a reasonable and comprehensive method is selected from the questionnaire method (PAQ, TTA, ARS, O*NET) which is easy to implement for data collection. In addition, this paper selects questionnaire as the construction method of the index system of cognitive ability, which also includes two reasons. Firstly, the index extraction of commander’s cognitive abilities is characterized by involving as many people as possible, involving different levels of people, avoiding conformity, realizing in a short period of time, collecting more details and quantifiable information. Secondly, the indicators of cognitive ability have not been collected in various fields, and the corresponding standards have not been formed. Meanwhile, the demands of cognitive ability vary with the requirements of tasks, and there are fewer references in other areas.

### 3.2 Selection of data acquisition method

Questionnaire survey method is a method to make a questionnaire through job analysis, and then extract the ability index by factor analysis on the basis of obtaining large sample data. The general procedure of questionnaire method includes the selection of subjects, the distribution of questionnaires, the collection of questionnaires, the analysis of questionnaires and the processing of results. In this paper, the Occupational Information Network (O*NET) questionnaire method was selected to collect commander cognitive ability data in order to extract commander ability indexes [18]. Because the O*NET job analysis system absorbs the advantages of a variety of job analysis tools (PAQ, CMQ, ARS), the evaluation project of its work ability dimension covers all the key items of the ARS questionnaire. O*NET’s research and development team is made up of the nation’s leading industrial and organizational psychologists and position analysts. The O*NET content model is built around six series of factors, namely Worker Characteristics, Worker Requirements, Experience Requirements, Occupational Requirements, Occupation-Specific Information and Workforce Characteristics. The cognitive ability studied in this paper is a three-level index of Worker Characteristics.

**Fig 2:**
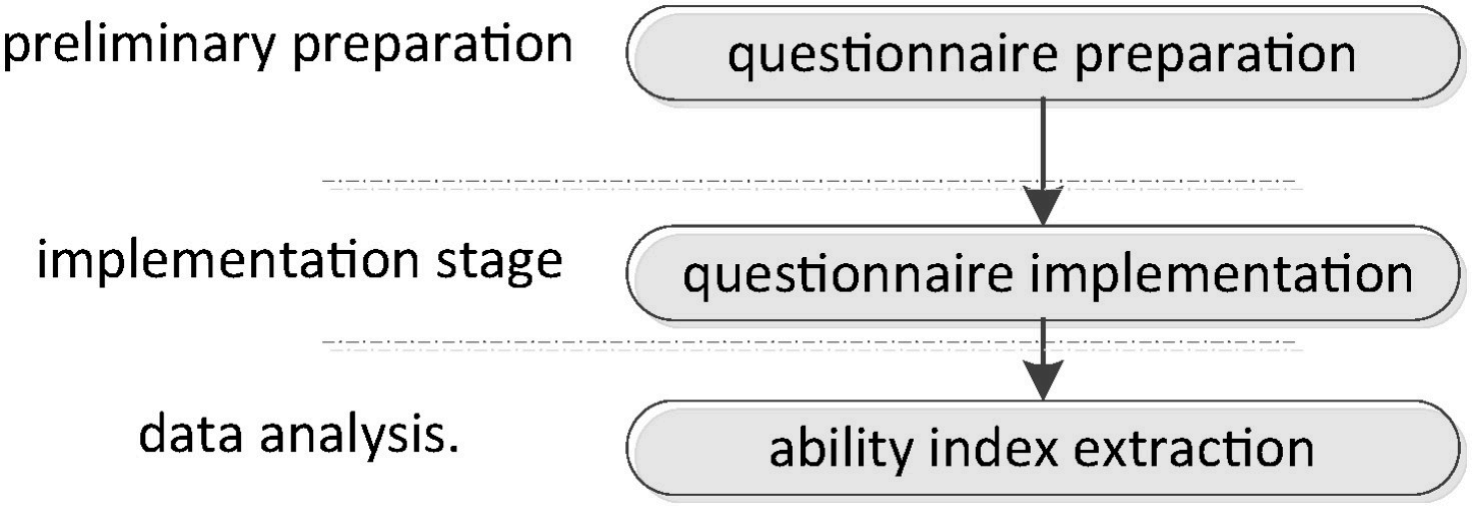
Implementation steps of the questionnaire method for extracting ability indexes.

The questionnaire method collected the importance evaluation data of commander’s cognitive ability indexes. The specific implementation steps for extracting core cognitive ability indicators are shown in Fig 2. Firstly, the commander questionnaire was completed and checked. Then the questionnaire survey and data collection were carried out. Finally, the important options affecting the commander’s cognitive ability were extracted from the questionnaire data through the reliability and validity test, and finally the indicator system of commander’s cognitive ability was obtained.

### 3.3 Questionnaire design and data collection

Considering that the purpose of this study is to extract the index system of cognitive ability of the commanders, the cognitive ability in O*NET ability dimension was selected as the foundation of the index system, which includes 7 abilities, namely verbal abilities, idea generation and reasoning abilities, quantitative abilities, memory ability and visual perception abilities, spatial reasoning, attention abilities. Through multiple interviews and surveys with commanders, the main tasks of ship commanders are divided into four aspects, including situation analysis, task planning, task command and comprehensive support. Therefore, the positions of commanders are also classified according to this classification. Situation analysis means that a ship commander pays attention to multiple targets on the ocean at any time during the execution of a mission, and judges the distance, azimuth, speed and threat level of each target from the ship. All 7 of these cognitive abilities are involved in this process. Task planning is to make corresponding plans according to the results of early situation analysis, such as slowing down, avoiding and reminding others. Cognitive skills involved in the task include idea generation and reasoning abilities, quantitative abilities, memory abilities. Task command refers to the process of task decomposition and execution in accordance with the task plan formulated in the early stage, during which the ship is constantly monitored, and the commander will see and check whether the ship is executed accurately according to the formulated plan. The cognitive abilities involved in this process include visual perception abilities, spatial reasoning abilities and attention abilities. Comprehensive support is to provide necessary support information for situation analysis, task planning and task command tasks (such as wind speed, wind direction and other meteorological information, oil, food surplus and other material information). All 7 of these cognitive abilities are involved in this process.

During the investigation and interviews, preliminary conclusions are found in the following aspects. Firstly, in the course of executing the mission, the commander needs to estimate the time when the target will arrive at the ship if it is close to the ship and has a high speed, so as to avoid ship collision. During this process, the commander often reviews the situation chart and the target information table Secondly, with the improvement of modern information system technology, the position of ships in the ocean can be accurately obtained through GPS signals, so that the relative position between different ships can be correctly displayed through the information system, so commanders rarely have the task of space positioning. Thirdly, during the execution of missions, commanders often perform rapid response tasks, such as quickly identifying whether a target is of high threat level or the depth of the ship relative to the sea bottom at the moment. Based on the above preliminary conclusions, the O*NET original scale was edited and adjusted. An index of graph comprehension was added in verbal ability. The visual summary speed in the visual perception ability index was revised into a frequently used time estimation index. The spatial positioning index in the spatial reasoning ability was deleted because this index corresponds to fewer work tasks. An important response ability was added, because this ability corresponds to more work tasks. After adjustment, only one index of spatial transformation is left in spatial reasoning ability. In the o* NET original scale, memory ability include one index and attention abilities include two. In order to facilitate data collection and analysis in the later stage, the two indexes of memory, spatial transformation and attention were combined and named mnemonic and attentive abilities. The adjusted O*NET questionnaire was formed through the above steps.

The final questionnaire includes verbal abilities (including oral understanding, oral expression, text understanding, graphic understanding, written expression total 5 indicators), idea generation and reasoning abilities (including fluency of ideas, originality, problem sensitivity, deductive reasoning, inductive reasoning, information ordering, category flexibility total 7 indicators), quantitative abilities (including mathematical reasoning, number flexibility 2 total indicators), visual perception abilities (including time valuation, visual search, perception speed total 3 indicators), mnemonic and attentive abilities (including working memory, spatial alternation, selective attention, degree of concentration total 4 indicators), response abilities (including simple response time, discriminative reaction time, selective reaction time total 3 indicators), 6 abilities a total of 23 indicators. Please refer to the corresponding table (S1 Table) for the abbreviations, definitions, and notations for the ability and corresponding indexes.

**Fig 3:**
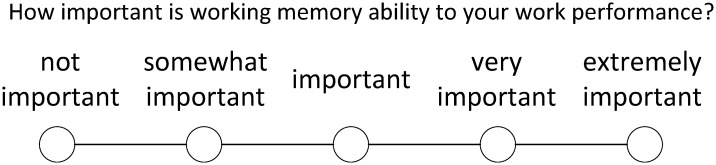
Ability questionnaire.

For each index, the questionnaire is precisely defined. For example, regarding the ability index of working memory (MAA1), the definition is the ability to memorize information, such as words, numbers, pictures, and programs. Commanders were asked to judge the importance of each indicator in the questionnaire, which was shown in Fig 3. For example, the question asked by the working memory (MAA1) index in the questionnaire is: *How important is working memory (MAA1) ability to the job performance of your position?* The questionnaire options are divided into not important, somewhat important, important, very important, and extremely important, corresponding to a score of 1-5. All indexes in this study were prepared according to this standard, please refer to the supporting information of this paper for detailed questionnaire contents (S1 File).

After completing the questionnaire, we invite skilled workers of various positions in the command and control system to participate in the questionnaire survey. All subjects were male commanders. The job context of these subjects is the cabin of the ship, which has been sailing on the sea for a long time. Their job positions are divided into 4 aspects, namely situation analysis, task planning, task command and comprehensive support. Professional titles are divided into two categories, one is the working age of more than five years (commonly referred to as professional commanders), the other is the working age of less than five years (commonly referred to as assistant commanders).

There are several things to be explain about ethical review of subjects. Firstly, the project is a research project that the ship commander management company needs to carry out. We are only participating in the project research as a technology provider. Secondly, the research in this paper only allow the subjects to score different questions, and does not cause any harm to the psychology and physiology of the subjects. Thirdly, in the early stage of project research, we have reminded the ship commander management company to apply to the corresponding ethics committee for ethical review. After the ship commander management company submitting the relevant ethical review work, they received a response from the local ethics committee “Research involving investigation or interview procedures, not involving potentially damaging and/or sensitive research in the subject can be exempted the process of ethical review.” This project was able to continue with the above conclusions. Fourthly, 2 points that need to be explained about the informed consent of the subjects. First of all, this experiment was carried out under the condition that the representatives of commanders were willing to participate in the experiment. Not all people need to participate. Secondly, we first obtained the oral consent of the commander and then read and explained the informed consent in person. The commander signed the informed consent after confirming the consent, and then filled out the questionnaire. Although the informed consent was signed, our questionnaires survey were answered under the condition of anonymity, which is to protect the commander’s privacy. Fifthly, the entire study is consistent with the principles expressed in the Declaration of Helsinki‘

When the questionnaire was sent out, the subject would give the commander a form S1 Table at the same time. Before answering the questionnaire, the subject will explain the cognitive ability and the meaning of the corresponding indexes to the commander in detail according to S1 Table, so as to ensure the commander can understand the meaning of the indexes accurately. After the commander understands the questionnaire, he can check the form S1 Table at any time in the process of answering. This questionnaire survey distributed 222 questionnaires to command and control system commanders, 20 of which were invalid and 202 were valid. The content of the questionnaire is invalid because the content is incomplete, and some questionnaires do not check the corresponding option.

### 3.4 Data preliminary analysis

#### 3.4.1 Results

In order to test the validity of the data, this paper makes a descriptive statistical analysis of the data, and the results are shown in Table 2. The mean value of all 24 indicators was greater than 2.5, so there were no indexes to be deleted. In general, the collected questionnaire data need to be analyzed for data reliability before further data processing, and the data reliability index is generally expressed by Cronbach’s core efficiency [24]. So, this paper carried out reliability analysis and calculated the Cronbach’s score coefficient. According to previous studies, if the Cronbach’s coefficient is greater than 0.7, it can be considered that the reliability between items is moderate [24]. Exploratory research, application research and scale development research can be conducted [24]. Cronbach’s coefficient > 0.8 can be applied to empirical studies [24]. Table 3 shows that the coefficient of cognitive ability is 0.735 and the coefficient based on the standardized item is 0.720. The coefficient of VA is greater than 0.7, and the coefficient of VPA, MMA, RA are all greater than 0.8. The coefficient of the above abilities indicates that the internal reliability of abilities can be explored and empiricism studied. However, the coefficient of IGRA and QA are 0.495 and 0.393 respectively, both of which are less than 0.7. The reliability coefficient is too low and the reliability is poor, so it is not suitable for exploratory factor analysis and confirmatory factor analysis. Therefore, they are deleted in the subsequent analysis.

**Table 2:** Item statistics.

CA	ICA	Mean	SD	N
VA	VA1	3.47	1.052	202
	VA2	3.21	0.834	202
	VA3	3.26	0.794	202
	VA4	3.17	0.786	202
	VA5	3.46	0.968	202
IGRA	IGRA1	2.98	0.846	202
	IGRA2	2.70	0.754	202
	IGRA3	3.21	1.022	202
	IGRA4	3.23	1.032	202
	IGRA5	2.72	0.755	202
	IGRA6	2.72	0.755	202
	IGRA7	3.00	0.838	202
QA	QA1	3.14	0.837	202
	QA2	3.14	0.687	202
VPA	VPA1	3.18	0.897	202
	VPA2	3.46	1.111	202
	VPA3	3.25	0.987	202
MAA	MAA1	3.25	1.258	202
	MAA2	3.24	1.190	202
	MAA3	3.32	1.229	202
	MAA4	3.38	1.192	202
RA	RA1	3.47	0.988	202
	RA2	3.43	0.981	202
	RA3	3.46	0.942	202

CA: Cognitive ability

ICA: Indexes of cognitive ability

SD: The standard deviation

N: Number of the data items

**Table 3:** Reliability statistics.

	Cronbach’s Alpha	Cronbach’s Alpha Based on Standardized Items	N
CA	0.735	0.720	24
VA	0.742	0.747	5
IGRA	0.495	0.512	7
QA	0.393	0.399	2
VPA	0.804	0.807	3
MAA	0.831	0.831	4
RA	0.818	0.818	3

CA: Cognitive ability

N: Number of the data items

#### 3.4.2 Discussion

There are two possible reasons for the low Cronbach’s coefficient and mean value of IGRA and QA. First, the commander group’s evaluation of the importance of cognitive ability of IGRA and QA is inconsistent. Some people have relatively high evaluation scores, while others have relatively low evaluation scores. From the column of mean in Table 2, we can see that the cognitive ability mean value of IGRA and QA is lower than other cognitive abilities on the whole, which also reflects that the commander group pays less attention to the cognitive ability of IGRA and QA on the whole. Secondly, cognitive processes corresponding to cognitive abilities of IGRA and QA are less frequent or difficult to perform tasks. If the task execution frequency corresponding to cognitive ability is low, the commander group will consider it as less important in the process of evaluation. If the task difficulty corresponding to cognitive ability is low, even if the frequency of occurrence is relatively high, the commander group will feel it is not important in the process of evaluation because of its low difficulty. The two factors of frequency and difficulty ultimately lead to a lower overall mean and poor consistency.

## 4 Data analysis

### 4.1 Exploratory factor analysis

#### 4.1.1 Results

In order to test the validity of the questionnaire and extract the indexes initially, we carried out exploratory factor analysis. The KMO and Bartlett’s Test and Total Variance Explained were calculated, corresponding to Tables 4 and 5. The Kaiser-Meyer-Olkin (KMO) Measure of Sampling Adequacy is a statistic that indicates the proportion of variance in your variables that might be caused by underlying factors. High values (close to 1.0) generally indicate that a factor analysis may be useful with your data. If the value is less than 0.50, the results of the factor analysis probably won’t be very useful [25]. The IBM SPSS 19.0 Statistic statistical analysis software was used in the calculation, factor analysis function was used, the correlation matrix option in descriptors was selected as KMO and Bartlett’s Test of sphericity, the method in rotation was selected as Varimax, and the rest were the software default options. Previous studies have suggested that KMO greater than 0.5 can be used for factor analysis, while KMO greater than 0.8 is very suitable for factor analysis [25]. The total value of Total Variance Explained should be between 50% and 90% for factor analysis [26]. According to the results of KMO and Bartlett’s Test, the determinant of cognitive ability is 0.005 greater than 0.0001. Meanwhile, the KMO value of cognitive ability is 0.8 greater than 0.5, and the KMO values of VA, VPA, MMA and RA are all greater than 0.5, which can be used for factor analysis. However, according to the results of Total Variance Explained, the Total Variance of verbal abilities is 49.751, less than 50%, which is not enough, and significantly less than the contribution of VPA, MMA, and RA to cognitive ability. From the data of contribution degree, it can be seen that although the commander group thinks that language ability is a part of cognitive ability, it is less important than other abilities. At the same time, considering the results of reliability analysis, Cronbach’s *α* standard coefficient is 0.747 less than 0.8, which is only suitable for exploratory research, but slightly lacking for empirical research. Therefore, the VA ability was finally deleted, and all indexes of the three abilities of VPA, MMA, and RA were re-calculated by KMO and Bartlett’s Test. The results are shown in Table 6. The determinant value was 0.016 greater than 0.0001, the KMO value was.832 greater than 0.8, the Chi-Square value was 808.218, the degree of freedom is 45, and the significance value was.000 less than 0.05, indicating that the data was significant and the result of factor analysis is reasonable.

**Table 4:** KMO and Bartlett’s test.

Cognitive ability	Kaiser-Meyer-Olkin Measure of Sampling Adequacy.		0.800
	Bartlett’s Test of Sphericity	Approx. Chi-Square	1046.661
		df	105
		*	0.000
VA	Kaiser-Meyer-Olkin Measure of Sampling Adequacy.		0.783
	Bartlett’s Test of Sphericity	Approx. Chi-Square	202.231
		df	10
		*	0.000
VPA	Kaiser-Meyer-Olkin Measure of Sampling Adequacy.		0.708
	Bartlett’s Test of Sphericity	Approx. Chi-Square	197.165
		df	3
		*	0.000
MAA	Kaiser-Meyer-Olkin Measure of Sampling Adequacy.		0.815
	Bartlett’s Test of Sphericity	Approx. Chi-Square	285.399
		df	6
		*	0.000
RA	Kaiser-Meyer-Olkin Measure of Sampling Adequacy.		0.713
	Bartlett’s Test of Sphericity	Approx. Chi-Square	210.533
		df	3
		*	0.000

“*” represents significant. When the value of significance is less than 0.05, it is indicated by *.

**Table 5:** Total variance explained.

Component	Initial Eigenvalues			Extraction Sums of Squared Loadings		
	Total	Variance %	Cumulative %	Total	Variance %	Cumulative %
Cognitive ability	4.275	28.499	28.499	4.275	28.499	28.499
	2.497	16.647	45.145	2.497	16.647	45.145
	1.448	9.656	54.802	1.448	9.656	54.802
	1.422	9.483	64.285	1.422	9.483	64.285
VA	2.488	49.751	49.751	2.488	49.751	49.751
	0.809	16.180	65.931			
	0.656	13.120	79.051			
	0.552	11.043	90.094			
	0.495	9.906	100.000			
VPA	2.166	72.188	72.188	2.166	72.188	72.188
	0.467	15.558	87.746			
	0.368	12.254	100.000			
MAA	2.655	66.381	66.381	2.655	66.381	66.381
	0.479	11.984	78.365			
	0.448	11.190	89.554			
	0.418	10.446	100.000			
RA	2.200	73.331	73.331	2.200	73.331	73.331
	0.446	14.857	88.187			
	0.354	11.813	100.000			

**Table 6:** KMO and Bartlett’s test.

Kaiser-Meyer-Olkin Measure of Sampling Adequacy.		832.
Bartlett’s Test of Sphericity	Approx. Chi—Square	808.218
	df	45
	*	000.

“*” represents significant. When the value of significance is less than 0.05, it is indicated by *.

In order to evaluate the intrinsic structure of the cognitive ability of the 10 indexes, principal component factor analysis was performed using the maximum variance rotation method. Because the purpose of this paper is to find the core indexes of multiple cognitive ability indexes. One of the functions of factor analysis rotation is that when identifying the representative factors of each principal component, it is easy to identify the index with larger weight in the principal component as the representative index of the principal component. The maximum variance rotation method is the most commonly used method in actual rotation, so this method is used. Three factors were obtained after rotation, among which the first factor accounted for 26.174% of the total variance, the second factor accounted for 22.554% of the total variance, and the third factor accounted for 21.946% of the total variance. Table 7 shows the indexes and factor loads of the rotation factor. For clear expression, the load value less than 0.5 is omitted.

**Table 7:** Rotated component matrix (a) and factor loading.

Ability	indicators	Component			Extraction
		1	2	3	
MAA	MAA2	0.818			681.
	MAA1	0.803			700.
	MAA3	0.757			669.
	MAA4	0.744			653.
RA	RA3		0.842		752.
	RA1		0.833		740.
	RA2		0.8		701.
VPA	VPA3			0.85	758.
	VPA2			0.826	679.
	VPA1			0.779	735.
The eigenvalue		2.617	2.255	2.195	
Variance contribution rate %		26.174	22.554	21.946	

Extraction Method: Principal Component Analysis.

Rotation Method: Varimax with Kaiser Normalization.

A. Rotation converged in 4 iterations.

#### 4.1.2 Discussion

The above statistical analysis shows that the final core cognitive abilities ere composed of MAA, RA and VPA. According to the variance contribution rate, the importance is basically equal. This reflects that the commander group thinks that these three cognitive abilities are very important and the importance is basically the same, which is also consistent with the situation analysis, task planning, task command and comprehensive support four typical task analysis contents in 3.3 section. Among them, MAA include MAA2, MAA1, MAA3 and MAA4, RA include RA3, RA1 and RA2, and VPA include VPA3, VPA2 and VPA1.

### 4.2 Confirmatory factor analysis

#### 4.2.1 Results

**Fig 4:**
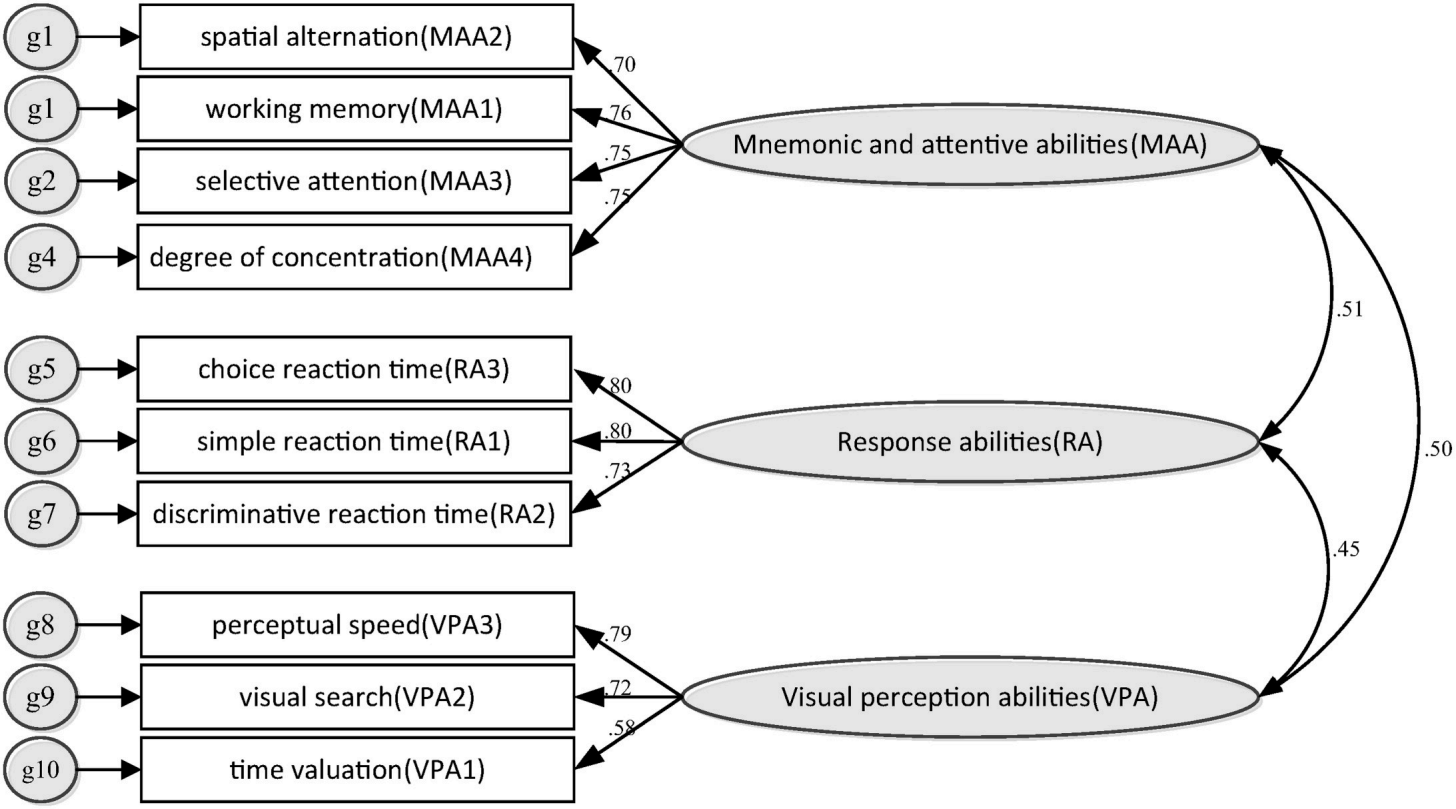
Structural model of cognitive ability factor and its standard solution.

In order to determine the accuracy of the extraction indexes of exploratory factor analysis, confirmatory factor analysis was carried out in this paper. This document uses AMOS 24 software for data processing, and the constructed cognitive factor structure model and its standard solution are shown in Fig 4. The model is divided into three abilities, namely MAA (including 4 indexes), RA (including 3 indexes), and VPA (including 3 indexes). The parameters of the model were set as follows. The “maximum likelihood” and “estimate means and intercepts” options under the “discrepancy parameter” in the “estimation” option were selected, the “standardized estimates” in the output option were selected, and the rest adopt the default options of the AMOS software.

In this paper, in order to determine the accuracy of the extraction indexes of exploratory factor analysis, CMIN/DF, GFI, CFI, TLI, RMSEA and RMR indexes were selected as the indexes of model test. Where, CMIN/DF is a statistic directly testing the similarity between the sample covariance matrix and the estimated variance matrix. Theoretically, the closer the value is to 1, the better the model fitting is [27]. The GFI index value is between 0 and 1, and the closer it is to 1, the better the fit is. It is generally believed that the standard of GFI is at least greater than 0.80 [28]. CFI index is obtained when comparing the hypothesis model and the independent model, and its value is between 0 and 1. The closer it is to 1, the better the fit is, and it is generally believed that CFI should be greater than 0.9 [23]. TLI index is a kind of comparative fitting index, its value is between 0 and 1. The closer it is to 1, the better the fit is. If TLI > 0.9, it is considered that the model fits well [28]. RMSEA is the index of evaluation model fitting. If it is close to 0, the fitting is well. It is generally believed that if RMSEA = 0, the model is completely fitted. RMSEA < 0.05 indicates that the model is close to fitting. 0.05 < RMSEA < 0.08 indicates that the model fitting is reasonable; 0.08 < RMSEA < 0.10, indicating general model fitting; RMSEA > 0.10, indicating poor model fitting [29];. RMR index measures the fitting degree of the model by measuring the average residual of the predicted correlation and the actual observation correlation. The closer it is to 0, the better the fit. If RMR < 0.1, it is considered that the model fits well [30].

Confirmatory factor analysis was carried out on the data samples, and the test index of the model is shown in Table 8. The path coefficients between the potential variables of the model are shown in Table 9.

**Table 8:** Results of the overall model fitness test for confirmatory factor analysis.

The dimension	CMIN/DF	GFI	CFI	TLI	RMSEA	RMR
Cognitive ability	1.222	0.965	0.991	0.987	0.033	0.045
Demonstrating compliance	Up to standard	Up to standard	Up to standard	Up to standard	Up to standard	Up to standard

**Table 9:** Path coefficients between potential variables of the model.

			Estimate	S.E.	C.R.	P	The Label
MAA2	←	MAA	1.000				
MAA1	←	MAA	1.025	104.	9.827	***	
MAA3	←	MAA	1.070	107.	9.991	***	
MAA4	←	MAA	925.	101.	9.205	***	
RA3	←	RA	1.000				
RA1	←	RA	1.097	113.	9.745	***	
RA2	←	RA	1.046	107.	9.748	***	
VPA3	←	VPA	1.000				
VPA2	←	VPA	738.	079.	9.312	***	
VPA1	←	VPA	890.	091.	9.818	***	

It can be concluded from Table 8 that CMIN/DF is 1.22 less than 3, GFI, CFI, and TLI are all greater than 0.9, and RMSEA and RMR are all less than 0.05, which indicating that the degree of model fitting is good, and it can be considered that the above cognitive ability index extraction results is feasible.

In Table 9, all variables in MMA, RA, and VPA abilities have a positive factor load on the corresponding ability, and the C.R. value is greater than 0.05, indicating that each variable has a significant impact on the corresponding ability. In MMA and RA, the importance of each variable to the factor load of ability is basically the same. In VPA, VP3 is larger than VP2, and VPA1 is slightly larger than VPA2. As can be seen from Table 10, the covariance between each ability variable is greater than 0, indicating that the change trend among the three abilities is the same, and C.R. is greater than 0.05, indicating that the changes among the various abilities affect each other significantly. It can be seen from Table 10 that the correlation values between the abilities are approximately equal t 0.5, with the same trend.

**Table 10:** Covariance and correlations between model abilities.

			Estimate	S.E.	C.R.	C. C	P	Label
MAA	↔	RA	0.325	0.066	4.892	0.505	***	
VPA	↔	MAA	0.391	0.079	4.920	0.500	***	
VPA	↔	RA	0.279	0.062	4.476	0.447	***	

C.C: correlation coefficients

#### 4.2.2 Discussion

The results of EFA and CFA data analysis can reflect the following conclusions. Firstly, the commander group believes that MMA, RA and VPA cognitive abilities are the core of the six cognitive abilities, and the importance of the three abilities is equal. Secondly, the results of data analysis are consistent with the content of cognitive process analysis of four typical tasks performed by commanders. Thirdly, for VPA ability, VPA1 is more important than VPA2 and VPA3, indicating that the commander will focus on the target that is coming to us and is relatively fast during the task execution process. Fourthly, the positive correlation between the variables also reflects that the commander group thinks that all the cognitive abilities are important.

### 4.3 Data analysis of working age

#### 4.3.1 Results

In order to study whether the professional commander and assistant commander have different evaluation on the ability and the importance of corresponding indexes. When collecting data, the subjects were divided into two categories: professional commander and assistant commander, and then the two types of data were analyzed. The reason for studying working age rather than actual age is that the ship’s commanders are all adults, and the time they take up their jobs are basically around 23 years old. In the actual work process, the total length of work of each commander is basically the same. The managed companies generally grant them different grades based on working age, so our research is based on working age. The minimum working age of all commander subjects is 2 years, and the maximum working age is 9 years. The independent sample T test is generally used to test whether there is a significant difference between two groups of data for the same target [31]. Therefore, the independent sample Levene T test for working age was performed on three abilities of MAA, RA, VPA, and the corresponding 10 indicators. The results are shown in Tables 11, 12 and 13.

**Table 11:** Group statistics.

	Work Experience	N	Mean	Std. Deviation	Std. Error scheme
MAA	Less than five years	140	3.143	1.004	0.085
	More than five years	62	3.645	0.875	0.111
RA	Less than five years	140	3.405	0.883	0.075
	More than five years	62	3.554	0.693	0.088
VPA	Less than five years	140	3.252	0.873	0.074
	More than five years	62	3.387	0.792	0.101

**Table 12:** Independent samples test (3 abilities).

		Levene’s Test for Equality of Variances		T-test for Equality of Means						
		F	Sig.	t	df	Sig. (2-tailed)	Mean Difference	Std. Error Difference	95% Confidence Interval of the Difference	
									The Lower	Upper
MAA	EVA	3.721	0.055	−3.406	200	0.001	−0.502	0.147	−0.793	−0.211
	EVNA			−3.592	133.108	0.000	−0.502	0.140	−0.779	−0.226
RA	EVA	3.403	0.067	−1.177	200	0.241	−0.149	0.127	−0.398	0.101
	EVNA			−1.291	146.872	0.199	−0.149	0.115	−0.377	0.079
VPA	EVA	0.016	0.899	−1.040	200	0.300	−0.135	0.130	−0.390	0.121
	EVNA			−1.080	128.055	0.282	−0.135	0.125	−0.382	0.112

EVA: Equal variances assumed

EVNA: Equal variances not assumed

**Table 13:** Independent samples test (10 indicators).

		Levene’s Test for Equality of Variances		T-test for Equality of Means						
		F	Sig	t	df	Sig. (2-tailed)	Mean Difference	Std. Error Difference	95% Confidence Interval of the Difference	
									The Lower	Upper
MAA2	EVA	2.030	0.156	−2.245	200	0.026	−0.404	0.180	−0.759	−0.049
	EVNA			−2.400	137.771	0.018	−0.404	0.168	−0.737	−0.071
MAA1	EVA	2.611	0.108	−3.801	200	0.000	−0.706	0.186	−0.073	−0.340
	EVNA			−3.762	114.152	0.000	−0.706	0.188	−0.078	−0.334
MAA3	EVA	0.209	0.648	−2.502	200	0.013	−0.448	0.179	−0.802	−0.095
	EVNA			−2.597	127.891	0.011	−0.448	0.173	−0.790	−0.107
MAA4	EVA	0.364	0.547	−2.431	200	0.016	−0.450	0.185	−0.816	−0.085
	EVNA			−2.506	125.773	0.013	−0.450	0.180	−0.806	−0.095
RA3	EVA	5.465	0.020	−1.209	200	0.228	−0.174	0.143	−0.456	0.109
	EVNA			−1.348	153.003	0.180	−0.174	0.129	−0.428	0.081
RA1	EVA	1.416	0.236	−1.260	200	0.209	−0.190	0.151	−0.486	0.107
	EVNA			−1.341	136.154	0.182	−0.190	0.141	−0.469	0.090
RA2	EVA	0.148	0.701	−0.560	200	0.576	−0.084	0.150	−0.379	0.212
	EVNA			−0.574	124.522	0.567	−0.084	0.146	−0.373	0.205
VPA3	EVA	0.070	0.792	−0.719	200	0.473	−0.108	0.151	−0.405	0.189
	EVNA			−0.732	122.320	0.465	−0.108	0.148	−0.401	0.184
VPA2	EVA	0.063	0.802	−1.356	200	0.177	−0.185	0.136	−0.454	0.084
	EVNA			−1.402	126.708	0.163	−0.185	0.132	−0.446	0.076
VPA1	EVA	0.029	0.866	−0.653	200	0.515	−0.111	0.170	−0.446	0.224
	EVNA			−0.653	117.003	0.515	−0.111	0.170	−0.447	0.225

EVA: Equal variances assumed

EVNA: Equal variances not assumed

Tables 12 and 13 are the standard results of the independent Levene T test output using SPSS software. In Tables 12 and 13, we generally focus on the second column, the fourth column (Sig.), and the seventh column (Sig. (2-tailed)). When the value of the Sig. column is greater than 0.05, we first locate “Equal variances assumed” row, and then view the value of the Sig. (2-tailed) column. If the value is less than 0.05, it means significant, if the value is greater than 0.05, it means not significant. When the value of the Sig. column is less than 0.05, we first locate the “Equal variances not assumed” row, and then look at the value of the Sig. (2-tailed) column. If the value is less than 0.05, it means significant, if the value is greater than 0.05, it means not significant.

As can be seen from Table 12, the significance (Sig. column) of Levene T test for the abilities of MAA, RA and VPA were all greater than 0.05. The first line “assuming equal variance” was used to explain and report the results of the T test. The result of the T test of MAA had a p value (Sig.(2-tailed) column) less than 0.05, indicating that there was a statistically significant difference in these skills between the professional commanders and assistant commanders. It can be seen from the results in Table 11 that subjects with the professional commanders all rated higher on the importance of these indicators than subjects with the assistant commanders. However, the Levene T test results of RA and VPA in Table 12 shows that the p value was greater than 0.05, indicating that there was no statistically significant difference between the professional commanders and assistant commanders in these skills. Furthermore, as can be seen from the results in Table 13, the p-value (Sig.(2-tailed) column) of the T test results of the four indicators of MAA were all less than 0.05, indicating that there was a statistically significant difference between the professional commanders and assistant commanders in these skills.

#### 4.3.2 Discussion

The results show that there are significant differences between assistant commanders and professional commanders in the evaluation of the importance of MAA ability, but there is no significant difference in the evaluation of RA and VPA. This may be due to the fact that the tasks corresponding to the four cognitive abilities contained in MAA are performed more frequently or more difficultly. The corresponding abilities of MMA were working memory (MMA1), spatial alternation (MMA2), selective attention (MAA3), and degree of concentration (MAA4). The above four abilities are often used in the process of situation analysis and task command. Taking working memory (MAA1) as an example, among the four abilities, working memory has the smallest calculation value of significance, which is reflected as the most obvious. The reasons are as follows. Working memory is generally divided into two types, short-term memory and long-term memory. In the task of situation awareness and task command, short-term memory generally corresponds to the temporary memory of the target. When there are many important targets, the commander’s temporary memory is required to be higher. The long-term memory generally corresponds to the high-risk and characteristic goals summarized for a long time, which will be accompanied by corresponding information. This kind of information needs long-term memory and can be recalled at any time in the process of task execution. When there are many goals, they also face the problem of memory pressure. These two aspects of memory are precisely what needs to be exercised most during work. Therefore, professional commanders generally rate MAA1’s importance more than assistant commanders.

### 4.4 Data analysis of two Bi-group of confirmatory factor analysis

#### 4.4.1 Results

In order to verify the stability of the structural model and the invariance of factors, this paper carried out two Bi-group confirmatory factor analysis. The first Bi-group confirmatory factor analysis is to randomly divide the data into two groups to measure the stability of the structural model. The scheme adopted in this paper is to divide 202 sets of data into two groups, the first group is a total of 101 data from 1 to 101, and the second group is the remaining 101 data. The second Bi-group confirmatory factor analysis is to divide the data into two groups of assistant commanders and professional commanders. The corresponding data numbers are 142 and 60. The AMOS software uses the same settings as 4.2 for confirmatory factor analysis. Tables 14 and 15 represent the analysis results of the first 101 data of the first Bi-group confirmatory factor analysis. Tables 16 and 17 represent the analysis results of the last 101 data of the first Bi-group confirmatory factor analysis. Tables 18, 19 and 20 represent the analysis results of 142 data in the second Bi-group verification analysis for assistant commanders. Tables 21, 22 and 23 represent the analysis results of 60 data in the second Bi-group verification analysis for professional commanders.

**Table 14:** Results of the overall model fitness test for confirmatory factor analysis (Numbers 1 through 101).

The dimension	CMIN/DF	GFI	CFI	TLI	RMSEA	RMR
Cognitive ability	1.230	0.930	0.978	0.969	0.048	0.054
Demonstrating compliance	Up to standard	Up to standard	Up to standard	Up to standard	Up to standard	Up to standard

**Table 15:** Path coefficients between potential variables of the model (Numbers 1 through 101).

			Estimate	S.E.	C.R.	P	The Label
MAA2	←	MAA	1.000				
MAA1	←	MAA	2.032	0.543	3.742	***	
MAA3	←	MAA	2.154	0.569	3.787	***	
MAA4	←	MAA	1.622	0.464	3.494	***	
RA3	←	RA	1.000				
RA1	←	RA	1.145	0.144	7.931	***	
RA2	←	RA	0.998	0.126	7.921	***	
VPA3	←	VPA	1.000				
VPA2	←	*VPA*	0.704	0.105	6.715	***	
VPA1	←	VPA	1.023	0.144	7.097	***	

**Table 16:** Results of the overall model fitness test for confirmatory factor analysis (Numbers 101 through 202).

The dimension	CMIN/DF	GFI	CFI	TLI	RMSEA	RMR
Cognitive ability	1.239	0.933	0.943	0.920	0.049	0.063
Demonstrating compliance	Up to standard	Up to standard	Up to standard	Up to standard	Up to standard	Up to standard

**Table 17:** Path coefficients between potential variables of the model (Numbers 101 through 202).

			Estimate	S.E.	C.R.	P	The Label
MAA2	←	MAA	1.000				
MAA1	←	MAA	−0.002	0.021	−0.104	0.917	par_1
MAA3	←	MAA	−0.001	0.013	−0.103	0.918	par_2
MAA4	←	MAA	−0.002	0.019	−0.103	0.918	par_3
RA3	←	RA	1.000				
RA1	←	RA	1.053	0.247	4.255	***	par_4
RA2	←	RA	1.109	0.260	4.258	***	par_5
VPA3	←	VPA	1.000				
VPA2	←	VPA	0.670	0.138	4.851	***	par_6
VPA1	←	VPA	0.815	0.149	5.477	***	par_7

**Table 18:** Item statistics of MMA (less than 5 years).

	MMA1	MMA2	MMA3	MMA4
Mean	3.04	3.10	3.18	3.26
N	140	140	140	140
Std. Deviation	1.208	1.207	1.242	1.237

**Table 19:** Results of the overall model fitness test for confirmatory factor analysis (less than 5 years).

The dimension	CMIN/DF	GFI	CFI	TLI	RMSEA	RMR
Cognitive ability	1.058	0.958	0.997	0.996	0.020	0.005
Demonstrating compliance	Up to standard	Up to standard	Up to standard	Up to standard	Up to standard	Up to standard

**Table 20:** Path coefficients between potential variables of the models (less than 5 years).

			Estimate	S.E.	C.R.	P	The Label
MAA2	←	MAA	1.000				
MAA1	←	MAA	0.922	0.108	8.556	***	
MAA3	←	MAA	0.942	0.105	8.999	***	
MAA4	←	MAA	0.862	0.105	8.208	***	
RA3	←	RA	1.000				
RA1	←	RA	1.143	0.127	8.966	***	
RA2	←	RA	1.113	0.125	8.941	***	
VPA3	←	VPA	1.000				
VPA2	←	VPA	0.792	0.092	8.618	***	
VPA1	←	VPA	0.907	0.102	8.901	***	

**Table 21:** Item statistics of MMA (more than 5 years).

	MMA1	MMA2	MMA3	MMA4
Mean	3.74	3.55	3.63	3.66
N	62	62	62	62
Std. Deviation	1.241	1.097	1.149	1.039

**Table 22:** Results of the overall model fitness test for confirmatory factor analysis (more than 5 years).

The dimension	CMIN/DF	GFI	CFI	TLI	RMSEA	RMR
Cognitive ability	0.974	0.915	1.000	1.009	0.000	0.063
Demonstrating compliance	Up to standard	Up to standard	Up to standard	Up to standard	Up to standard	Up to standard

**Table 23:** Path coefficients between potential variables of the models (more than 5 years).

			Estimate	S.E.	C.R.	P	The Label
MAA2	←	MAA	1.000				
MAA1	←	MAA	1.401	0.350	4.002	***	
MAA3	←	MAA	1.437	0.366	3.928	***	
MAA4	←	MAA	1.141	0.308	3.700	***	
RA3	←	RA	1.000				
RA1	←	RA	0.852	0.227	3.747	***	
RA2	←	RA	0.749	0.199	3.761	***	
VPA3	←	VPA	1.000				
VPA2	←	VPA	0.544	0.147	3.691	***	
VPA1	←	VPA	0.828	0.203	4.088	***	

#### 4.4.2 Discussion

The conclusion of the first Bi-group confirmatory factor analysis experiment is divided into the following two aspects. Firstly, judging from the overall fitness test results of the model, although the CMIN/DF, GFI, CFI, TLI, RMSEA and RMR of the two models are slightly inferior to Table 8, the fitting effect is also very good. Secondly, from the results of the model’s potential variable path coefficients, the contribution of various cognitive ability indexes in the model to cognitive ability is not as stable as in Table 8, showing a certain degree of fluctuation, which may be due to the sample size reduced by half. The conclusion of the second Bi-group confirmatory factor analysis experiment is divided into the following three aspects: Firstly, for the index of the independent T test ability that showed significant MAA ability, the average score of professional commanders is higher than that of assistant commanders., Which also led to its significance for working age. Secondly, the CMIN/DF, GFI, CFI, TLI, RMSEA, and RMR of the two models are basically the same as those in Table 8, the fit is even better. Thirdly, as can be seen from Tables 20 and 23, for MAA cognitive ability, the contribution of professional commanders’ MAA index is greater than that of assistant commanders, which also verifies the difference in the evaluation of commander groups. The above conclusions indicate that the structural model has good stability and factor invariance.

## 5 Conclusion

The measurement of cognitive ability is mainly used for the design of the human-machine interface information and interaction design of the command and control system to ensure that the requirements for the commander to complete the operation task will not exceed the commander’s ability limit and match the commander’s cognitive law. Based on the typical o* NET questionnaire in the world, this paper makes a simple revision of the o* NET questionnaire and adds some indexes such as reaction ability. It makes a questionnaire to collect the importance evaluation of cognitive ability of command and control system commanders, and collects the complete questionnaire data of 202 commanders. 202 representatives are selected from 4 positions of the command and control system of the ship to collect data. The results can reflect the evaluation level of the overall cognitive ablity index system.

Through the data preliminary analysis, exploratory factor analysis, confirmatory factor analysis, independence T test, and two Bi-group of confirmatory factor analysis of the collected data, the final conclusions are as follows. Firstly, the most important cognitive abilities that affect command and control system commanders are visual perception abilities (VPA) (including time valuation (VPA1), visual search (VPA2), perception speed (VP3) total 3 indexes), mnemonic and attentive abilities (MAA) (including working memory (MAA1), spatial alternation (MAA2), selective attention (MAA3), degree of concentration (MAA4) total 4 indexes), response abilities (RA) (including simple response time (RA1), discriminative reaction time (RA2), selective reaction time (RA3) total 3 indexes). Verbal abilities (VA), idea generation and reasoning abilities (IGRA), quantitative abilities (QA) are relatively unimportant. Secondly, in the results of confirmatory factor analysis, CMIN/DF, GFI, CFI, TLI, RMSEA, RMR and other indicators used in the model test are all meet the requirements. The fitting degree of the model is good, and the whole index extraction method is feasible. Thirdly, the independence T test results show that for assistant commanders and professional commanders, there was a significant difference in the importance evaluation of mnemonic and attentive abilities (MAA), while there was no significant difference in the importance evaluation of response abilities (RA) and visual perception abilities (VPA). This difference is related to the frequency and difficulty of performing tasks corresponding to cognitive ability. We have analyzed in detail the reasons for working memory. Finally, the two Bi-group of confirmatory factor analysis shows that the structural model has good stability and factor invariance.

Through the research of this paper, the index system which can express the core cognitive ability of the commander of command and control system is successfully constructed, and the index system has been fully verified by mathematics. The 3 abilities and 10 indexes in the index system are closely related to the work tasks of operators, which also reflects the correctness of our construction results to a certain extent. According to the results of data analysis, there are differences between assistant commanders and professional commanders in the evaluation of the importance of some indexes, which reflects the importance of working age and experience to the promotion of position skills. The results of this research are of great significance for the subsequent acquisition of cognitive ability data and assessment of post cognitive ability of command and control system commanders.

## Supporting information

S1 Fig The score of the indexes of MAA, VPA, RA. (TIF)

S1 Table The abbreviations, definitions, and notations for the ability and corresponding items. (PDF)

S2 Table The variance matrices of KMO and Bartlett’s Test. (PDF)

S1 File The english version of this questionnaire. (PDF)

S2 File The original language version of this questionnaire. (PDF)
