# Supplementary figures and images for: Construction and confirmatory factor analysis of the core cognitive ability index system of ship C2 system operators
Source: PLoS One. 2020 Aug 24;15(8):e0237339. doi: 10.1371/journal.pone.0237339 (PMC7446803; doi:10.1371/journal.pone.0237339)

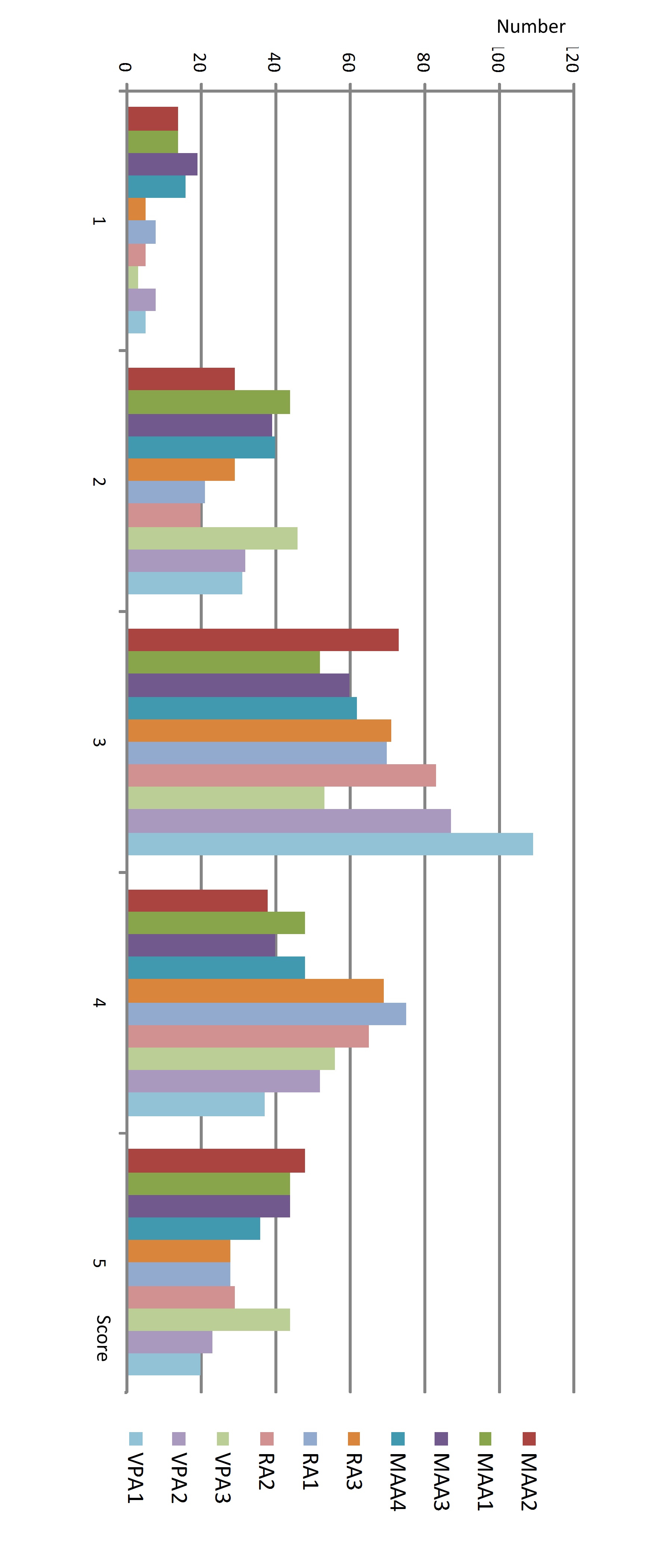

Supplement: S1 Fig — (TIF) [file pone.0237339.s001.tif]
