# Supplementary material for: Construction and confirmatory factor analysis of the core cognitive ability index system of ship C2 system operators
Source: PLoS One. 2020 Aug 24;15(8):e0237339. doi: 10.1371/journal.pone.0237339 (PMC7446803; doi:10.1371/journal.pone.0237339)
Supplement: S1 Table — (PDF) [file pone.0237339.s002.pdf]

Cognitive ability(abbreviation)	definitions	Index of cognitive ability(notations)	definitions
verbal abilities(VA)	Abilities that influence the acquisition and application of verbal information in problem solving	oral understanding(VA1)	The ability to listen to and understand information and ideas presented through spoken words and sentences
		oral expression(VA2)	The ability to communicate information and ideas in speaking so others will understand
		text understanding(VA3)	The ability to read and understand information and ideas presented in writing
		graphic understanding(VA4)	The ability to read and understand information and ideas presented in graphic
		written expression(VA5)	The ability to communicate information and ideas in writing so others will understand
Idea generation and reasoning abilities(IGRA)	Abilities that influence the application and manipulation of information in problem solving	fluency of ideas(IGRA)	The ability to come up with unusual or clever ideas about a given topic or situation, or to develop creative ways to solve a problem
		originality(IGRA1)	The ability to tell when something is wrong or is likely to go wrong It does not involve solving the problem, only recognizing there is a problem
		problem sensitivity(IGRA2)	The ability to apply general rules to specific problems to produce answers that make sense
		deductive reasoning(IGRA3)	The ability to combine pieces of information to form general rules or conclusions (includes finding a relationship among seemingly unrelated events)
		inductive reasoning(IGRA4)	The ability to arrange things or actions in a certain order or pattern according to a specific rule or set of rules (e.g., patterns of numbers, letters, words, pictures, mathematical operations)
		information ordering(IGRA5)	The ability to generate or use different sets of rules for combining or grouping things in different ways
		category flexibility(IGRA6)	The ability to come up with unusual or clever ideas about a given topic or situation, or to develop creative ways to solve a problem

Cognitive ability(abbreviation)	definitions	Index of cognitive ability(notations)	definitions
Quantitative abilities(QA)	Abilities that influence the solution of problems involving mathematical relationships	mathematical reasoning(QA1)	The ability to choose the right mathematical methods or formulas to solve a problem
		number flexibility(QA2)	The ability to add, subtract, multiply, or divide quickly and correctly.
Visual perception abilities(VPA)	Abilities related to the acquisition and organization of visual information	time valuation(VPA1)	The ability to estimate time spent or time that will be needed in the future
		visual search(VPA2)	The ability to identify or detect a known pattern (a figure, object, word, or sound) that is hidden in other distracting material
		perceptual speed(VPA3)	The ability to quickly and accurately compare similarities and differences among sets of letters, numbers, objects, pictures, or patterns The things to be compared may be presented at the same time or one after the other This ability also includes comparing a presented object with a remembered object
Mnemonic and attentive abilities(MAA)	Abilities related to memory and attention	working memory(MAA1)	The ability to remember information such as words, numbers, pictures, and procedures
		spatial alternation(MAA2)	The ability to imagine how something will look after it is moved around or when its parts are moved or rearranged
		selective attention(MAA3)	The ability to shift back and forth between two or more activities or sources of information (such as speech, sounds, touch, or other sources)
		degree of concentration(MAA4)	The ability to concentrate on a task over a period of time without being distracted
Response abilities (RA)	Abilities related to Response	simple reaction time(RA1)	The ability to quickly respond (with the hand, finger, or foot) to a signal (sound, light, picture) when it appears
		discrimination reaction	The ability to quickly respond (with the hand, finger, or foot) to a specific target

Cognitive ability(abbreviation)	definitions	Index of cognitive ability(notations)	definitions
		time(RA2)	signal (sound, light, picture) when it appears(such as determining whether the current goal is the one you want)
		selective reaction time(RA3)	The ability to quickly respond (with the hand, finger, or foot) to a different target signal (sound, light, picture) when it appears (such as determining which goal in the goal set the current goal is)
