# Supplementary material for: Construction and confirmatory factor analysis of the core cognitive ability index system of ship C2 system operators
Source: PLoS One. 2020 Aug 24;15(8):e0237339. doi: 10.1371/journal.pone.0237339 (PMC7446803; doi:10.1371/journal.pone.0237339)
Supplement: S2 Table — (PDF) [file pone.0237339.s003.pdf]

	Component			
	1	2	3	4
VA1	-.121	.723	.027	.008
VA2	-.109	.726	.111	-.111
VA3	-.079	.717	.126	.212
VA4	-.069	.666	-.022	.014
VA5	-.108	.649	-.163	-.005
VPA1	.635	.010	.003	-.530
VPA2	.616	.166	.130	-.558
VPA3	.605	.084	.091	-.609
MMA1	.688	.062	-.441	.131
MMA2	.603	.126	-.503	.222
MMA3	.705	.050	-.303	.272
MMA4	.724	.019	-.334	.141
RA1	.633	.036	.500	.291
RA2	.649	-.075	.470	.232
RA3	.629	.062	.494	.321

Extraction Method: Principal Component Analysis.

a. 4 components extracted.
