# Supplementary material for: Construction and confirmatory factor analysis of the core cognitive ability index system of ship C2 system operators
Source: PLoS One. 2020 Aug 24;15(8):e0237339. doi: 10.1371/journal.pone.0237339 (PMC7446803; doi:10.1371/journal.pone.0237339)
Supplement: S1 File — (PDF) [file pone.0237339.s004.pdf]

Name		Age		Sex	
Job position		Working age		Professional title	
Verbal abilities	How important is oral understanding ability to the job performance of your position?				
	not important (1)	somewhat important(2)	important(3)	very important(4)	extremely important(5)
	How important is oral expression ability to the job performance of your position?				
	not important (1)	somewhat important(2)	important(3)	very important(4)	extremely important(5)
	How important is text understanding ability to the job performance of your position?				
	not important (1)	somewhat important(2)	important(3)	very important(4)	extremely important(5)
	How important is graphic understanding ability to the job performance of your position?				
	not important (1)	somewhat important(2)	important(3)	very important(4)	extremely important(5)
	How important is written expression ability to the job performance of your position?				
	not important (1)	somewhat important(2)	important(3)	very important(4)	extremely important(5)
Idea generation and reasoning abilities	How important is Fluency of ideas ability to the job performance of your position?				
	not important (1)	somewhat important(2)	important(3)	very important(4)	extremely important(5)
	How important is originality ability to the job performance of your position?				
	not important (1)	somewhat important(2)	important(3)	very important(4)	extremely important(5)
	How important is problem sensitivity ability to the job performance of your position?				
	not important (1)	somewhat important(2)	important(3)	very important(4)	extremely important(5)
	How important is deductive reasoning ability to the job performance of your position?				
	not important (1)	somewhat important(2)	important(3)	very important(4)	extremely important(5)
	How important is inductive reasoning ability to the job performance of your position?				

	not important (1)	somewhat important(2)	important(3)	very important(4)	extremely important(5)
	How important is information ordering ability to the job performance of your position?				
	not important (1)	somewhat important(2)	important(3)	very important(4)	extremely important(5)
	How important is Category flexibility ability to the job performance of your position?				
	not important (1)	somewhat important(2)	important(3)	very important(4)	extremely important(5)
Quantitative abilities	How important is Mathematical reasoning ability to the job performance of your position?				
	not important (1)	somewhat important(2)	important(3)	very important(4)	extremely important(5)
	How important is number flexibility ability to the job performance of your position?				
	not important (1)	somewhat important(2)	important(3)	very important(4)	extremely important(5)
Visual perception abilities	How important is Time valuation ability to the job performance of your position?				
	not important (1)	somewhat important(2)	important(3)	very important(4)	extremely important(5)
	How important is Visual search ability to the job performance of your position?				
	not important (1)	somewhat important(2)	important(3)	very important(4)	extremely important(5)
	How important is Perceptual speed ability to the job performance of your position?				
	not important (1)	somewhat important(2)	important(3)	very important(4)	extremely important(5)
Mnemonic and attentive abilities	How important is Working memory ability to the job performance of your position?				
	not important (1)	somewhat important(2)	important(3)	very important(4)	extremely important(5)
	How important is Spatial alternation ability to the job performance of your position?				
	not important (1)	somewhat important(2)	important(3)	very important(4)	extremely important(5)
	How important is Selective attention ability to the job performance of your position?				

	not important (1)	somewhat important(2)	important(3)	very important(4)	extremely important(5)
	How important is Degree of concentration ability to the job performance of your position?				
	not important (1)	somewhat important(2)	important(3)	very important(4)	extremely important(5)
Response abilities	How important is Simple reaction time ability to the job performance of your position?				
	not important (1)	somewhat important(2)	important(3)	very important(4)	extremely important(5)
	How important is Discrimination reaction time ability to the job performance of your position?				
	not important (1)	somewhat important(2)	important(3)	very important(4)	extremely important(5)
	How important is Selective reaction time ability to the job performance of your position?				
	not important (1)	somewhat important(2)	important(3)	very important(4)	extremely important(5)
