# Supplementary material for: Construction and confirmatory factor analysis of the core cognitive ability index system of ship C2 system operators
Source: PLoS One. 2020 Aug 24;15(8):e0237339. doi: 10.1371/journal.pone.0237339 (PMC7446803; doi:10.1371/journal.pone.0237339)
Supplement: S2 File — (PDF) [file pone.0237339.s005.pdf]

姓名		年龄		性别	
工作岗位		工作年龄		职称	
	您觉得口语理解能力对您当前的工作有多重要？				
	不重要（1）	有点重要（2）	重要（3）	非常重要（4）	极其重要（5）
	您觉得口语表达能力对您当前的工作有多重要？				
	不重要（1）	有点重要（2）	重要（3）	非常重要（4）	极其重要（5）
	您觉得文本理解能力对您当前的工作有多重要？				
语言能力	不重要（1）	有点重要（2）	重要（3）	非常重要（4）	极其重要（5）
	您觉得图表理解能力对您当前的工作有多重要？				
	不重要（1）	有点重要（2）	重要（3）	非常重要（4）	极其重要（5）
	您觉得书面表达能力对您当前的工作有多重要？				
	不重要（1）	有点重要（2）	重要（3）	非常重要（4）	极其重要（5）
	您觉得思维的流畅性能力对您当前的工作有多重要？				
	不重要（1）	有点重要（2）	重要（3）	非常重要（4）	极其重要（5）
	您觉得创意能力对您当前的工作有多重要？				
	不重要（1）	有点重要（2）	重要（3）	非常重要（4）	极其重要（5）
	您觉得问题敏感度能力对您当前的工作有多重要？				
	不重要（1）	有点重要（2）	重要（3）	非常重要（4）	极其重要（5）
	您觉得演绎推理能力对您当前的工作有多重要？				
创意和推理能力	不重要（1）	有点重要（2）	重要（3）	非常重要（4）	极其重要（5）
	您觉得归纳推理能力对您当前的工作有多重要？				
	不重要（1）	有点重要（2）	重要（3）	非常重要（4）	极其重要（5）
	您觉得信息排序能力对您当前的工作有多重要？				
	不重要（1）	有点重要（2）	重要（3）	非常重要（4）	极其重要（5）
	您觉得分类的灵活性能力对您当前的工作有多重要？				
	不重要（1）	有点重要（2）	重要（3）	非常重要（4）	极其重要（5）
	您觉得数学推理能力对您当前的工作有多重要？				
数学能力	不重要（1）	有点重要（2）	重要（3）	非常重要（4）	极其重要（5）
	您觉得数学灵活性能力对您当前的工作有多重要？				
	不重要（1）	有点重要（2）	重要（3）	非常重要（4）	极其重要（5）
	您觉得时间估计能力对您当前的工作有多重要？				

视觉感知能力	不重要（1）	有点重要（2）	重要（3）	非常重要（4）	极其重要（5）
	您觉得视觉搜索能力对您当前的工作有多重要？				
	不重要（1）	有点重要（2）	重要（3）	非常重要（4）	极其重要（5）
	您觉得知觉速度能力对您当前的工作有多重要？				
	不重要（1）	有点重要（2）	重要（3）	非常重要（4）	极其重要（5）
意力与记忆能	您觉得工作记忆能力对您当前的工作有多重要？				
	不重要（1）	有点重要（2）	重要（3）	非常重要（4）	极其重要（5）
	您觉得空间变换能力对您当前的工作有多重要？				
	不重要（1）	有点重要（2）	重要（3）	非常重要（4）	极其重要（5）
	您觉得注意力广度能力对您当前的工作有多重要？				
	不重要（1）	有点重要（2）	重要（3）	非常重要（4）	极其重要（5）
	您觉得注意力集中程度能力对您当前的工作有多重要？				
	不重要（1）	有点重要（2）	重要（3）	非常重要（4）	极其重要（5）
反应能力	您觉得简单反应时能力对您当前的工作有多重要？				
	不重要（1）	有点重要（2）	重要（3）	非常重要（4）	极其重要（5）
	您觉得辨别反应时能力对您当前的工作有多重要？				
	不重要（1）	有点重要（2）	重要（3）	非常重要（4）	极其重要（5）
	您觉得选择反应时能力对您当前的工作有多重要？				
	不重要（1）	有点重要（2）	重要（3）	非常重要（4）	极其重要（5）
